# Droplet microfluidics integrated with machine learning reveals how adipose-derived stem cells modulate endocrine response and tumor heterogeneity in ER^+^ breast cancer†

**DOI:** 10.1039/d5lc00320b

**Published:** 2025-06-13

**Authors:** Braulio Andrés Ortega Quesada, Calley Chauvin, Elizabeth Martin, Adam Melvin

**Affiliations:** a Department of Chemical and Biomolecular Engineering, Clemson University Clemson SC 29634 USA melvina@clemson.edu; b Department of Biological and Agricultural Engineering, Louisiana State University Baton Rouge LA 70803 USA; c Department Medicine, Section Hematology and Medical Oncology, Tulane University New Orleans LA 70118 USA emartin1@tulane.com

## Abstract

Approximately 70% of breast cancer (BC) diagnoses are estrogen receptor positive (ER^+^) with ∼40% of ER^+^ BC patients presenting *de novo* resistance to endocrine therapy (ET). Recent studies identify the tumor microenvironment (TME) as having a key role in endocrine resistance in which adipose-derived stem cells (ASCs) play an essential role in cancer progression. Prior studies have indicated that ASC characteristics such as age and BMI may play a role in cancer progression. Unfortunately, most studies on ASC-BC cross talk have relied on established two-dimensional (2D) culture systems or the use of conditioned media that cannot replicate the complexity of the three-dimensional (3D) environment. This study used a microfluidic droplet trapping array and thiol–acrylate (TA) hydrogel scaffold to co-culture ER^+^ BC cells and ASCs as individual 3D spheroids (single culture) or organoids (co-culture) in a single device. Endocrine response was interrogated in both spheroids and organoids through the evaluation of proliferation following treatment with the selective estrogen receptor degrader (SERD) fulvestrant (ICI 182 780) followed by 17β-estradiol (E2). Terminal immunostaining for the proliferation marker (Ki67) was performed to evaluate how the presence of ASCs from different donor backgrounds (age and BMI) can modulate endocrine response. Results demonstrated that organoids containing two model ER^+^ cell lines (MCF7 and ZR-75) exhibited enhanced Ki67 expression even in the presence of ICI, suggesting a role for ASCs in cancer progression and endocrine resistance. Data clustering and classification algorithms were employed to categorize cellular behavior based on Ki67 expression and spheroid area to identify distinct clusters with high (H), intermediate (I), and low (L) Ki67 expression. Machine learning further stratified the data and revealed the direct effects of ASCs on Ki67 expression as well as how donor-specific features influenced ASC-driven changes in the TME. Notably, ASCs from an aged donor (>50) with lower BMI (<30) were able to enhance Ki67 expression even in the presence of endocrine therapy, while younger (<40) donors substantially enhanced Ki67 expression in the absence of both ICI and E2. Together, this study demonstrates the utility/development of a biomimetic culture system that recreates heterogenic 3D ER^+^ tumors through the co-culture of cancer cells with ASCs. This system provided insight into cell-extrinsic factors that govern ER^+^ breast cancer heterogeneity and response to endocrine therapy can be gained.

## Abstract 2

About 40% of breast cancer patients treated with endocrine therapy develop resistance at some point. It is suspected that stromal cells and cell heterogeneity play a key role in this process.

## Graphical abstract

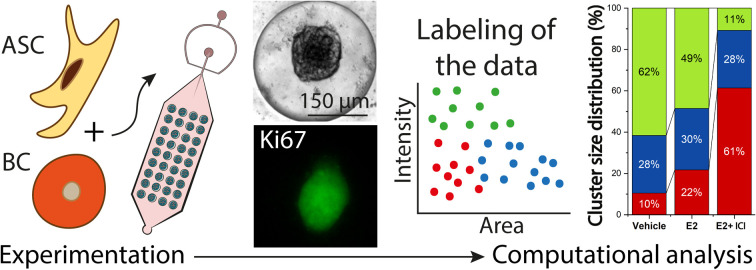

## Introduction

Breast cancer (BC) remains one of the most common cancers in the United States with ∼70% of new diagnosis being classified as estrogen receptor positive (ER^+^).^1^ The initial neo-adjuvant treatment strategy for ER^+^ BC is endocrine therapy (ET) which prevents estrogen synthesis or binding; however, ∼40% of the patients have *de novo* or acquired resistance to therapy with the underlying cause still not well understood.^2^ The hormone receptor classification in BC is useful in terms of treatment guidance and prognostic indicator, despite this BC still remains a highly complex and heterogenous disease. The heterogeneity of cancer cells between patients and within the tumor itself represents a significant barrier to establishing prognosis and treatment.^3^ This heterogeneous nature of cancer can be attributed to the presence of distinct subpopulations of cells that present differences in their metastatic potential, therapeutic sensitivities, and tumorigenicity.^4–6^ Additionally, the breast tumor microenvironment (TME) is a complex, diverse system consisting of infiltrating cells (*e.g.*, stem cells, stromal cells, and immune cells) that communicate with cancer cells *via* paracrine signaling molecules and matrix.^7,8^ This cell-to-cell communication coupled with cell-to-matrix communication through extracellular matrix (ECM) adhesion has been suggested to drive cancer proliferation, drug resistance, and enhanced metastasis.^9^ Elucidating cancer-stromal cell cross talk has the potential to provide new insight and understanding of tumorigenesis and drug resistance.^10^ Specifically, adipose derived stem cells (ASCs) upregulate signaling factors, matrix metalloproteases (MMPs), and secrete cytokines that play a role in tumor initiation, proliferation, metastasis, and angiogenesis.^11–17^ Furthermore, the manner in which ASCs impact breast cancer cells has been linked to ASC phenotype, with differences observed based on donor characteristics that may contribute to tumor heterogeneity.^18,19^ Specifically, patient age and BMI (or obesity) have been identified as two major factors that may affect ASC influence on breast cancer cells and cancer progression.^20–22^ Prior studies on breast cancer demonstrate the potential for the TME to impact breast cancer outcomes, specifically it has been observed that both age and weight/BMI impact prognosis and survival in breast cancer.^23–26^ Resident cells in the adipose tissue environment around breast tumors may be candidate cells for the observed differences in outcome. Stem cell function and cytokine profile is specified by both the tissue of origin (bone, adipose) and donor demographic (BMI, age).^27–30^ Prior studies on stem cell-mediated regulation of breast cancer phenotype have demonstrated the ability of these cells to mediated breast cancer biology based on donor demographic, thus motivating the need for advanced co-culture models.^21,22,31^

While some studies have implicated ASCs as key mediators of the TME, there is still much that is unknown on how ASCs interact with ER^+^ BC. This inability to fully define ASC-BC cross talk can be attributed to the current methods used to study cell-to-cell communication. The most common approaches include using conditioned media (CM), a Transwell assay, or two-dimensional (2D) direct mixed culture. Trivanović *et al.* showed that normal ASCs, BC-adjacent ASCs, and non-BC-adjacent ASCs all induced proliferation on MCF7 cells.^32^ Sabol *et al.* found that the 2D co-culture of ASCs with MCF7 and ZR-75 cells caused a decrease in the apoptotic index and increase in the survival fraction after cancer cells were exposed to radio therapy with the effect being more pronounced with obese *versus* lean ASCs.^33^ Using CM, differences in the ability of young and aged ASCs to induce proliferation and activate ER signaling in MCF7 cells was identified.^20^ While promising in providing insight into how ASCs affect cancer progression, these studies were limited in their ability to recapitulate the complex cell-to-cell and cell-to-matrix interactions observed in the TME by only using 2D culture approaches. Recent efforts have aimed to overcome this limitation using 3D spheroids as a promising approach to bridge the gap between 2D *in vitro* culture and *in vivo* models. Cancer spheroids closely mimic tumor features such as growth kinetics, metabolic rates, cell-to-cell interactions, and resistance to treatments.^34–36^ Spheroids have also been utilized for stromal/stem cells where Skiles *et al.* made ASC spheroids by aggregation and cultured the spheroids in 2% or 20% oxygen to evaluate VEGF secretion. Luo *et al.* developed a method to form 3D ASC spheroids based on a self-feeder layer (SLF) and compared their proliferation with spheroids using the ultra-low attachment surface. Other studies have tried to create ASC spheroids using microfluidic techniques as it is a powerful tool to control the uniformity and reproducibility of the spheroids, along with high throughput capabilities to index hundreds of spheroids to account for intratumor heterogeneity. Mesquita *et al.* used a continuous process for the encapsulation of mesenchymal stem cell spheroids into alginate hydrogel droplets using a glass-capillary-based microfluidic device.^37^ 3D organoids have been shown to be the next generation of spheroid approaches to more closely mimic the *in vivo* TME. Unlike spheroids, organoids are classified by organ-specific cells derived from primary tissue grown in a 3D matrix with other cell types and can mimic the pathophysiology of tumors.^38^ Some studies have incorporated ASCs with other cell types in organoids. Watzling *et al.* cultured MDA-MB-231 and MCF7 BC cells and ASC spheroids in agarose molds in direct contact to evaluate the expression of C–C motif chemokine ligand 5 (CCL5) and receptor C–C chemokine receptor type 1 (CCR1).^58^ Unfortunately, none of the prior studies have addressed the role of ASCs regulation of endocrine response in ER^+^ breast cancer using a 3D co-culture model. Moreover, these prior studies have not accounted for the heterogeneity between ASC donors.

In this study, ER^+^ BC monocultured spheroids (herein referred as MCF7 or ZR-75 spheroids) and organoids composed of primary ASCs co-cultured with ER^+^ cell lines (herein referred to as MCF7 or ZR-75 organoids) were 3D cultured through a previously developed indexable microfluidic droplet generator and trapping array coupled with a thiol acrylate (TA) hydrogel scaffold.^39,40^ This allowed for the culture of ∼450 spheroids or organoids in a single device with three devices being run in parallel to study intra-tumor heterogeneity. ASCs were isolated from three female donors with varying age and BMI to address inter-tumor heterogeneity. The proliferative response was assessed under different treatment conditions where spheroids or organoids were cultured in the presence or absence of a selective estrogen receptor degrader (SERD), Fulvestrant (also referred to as ICI 182 780) and then exogenous estrogen 17β-estradiol (E2), to evaluate the impact of ASCs on endocrine response. For the sake of clarity, Fulvestrant treatment will herein be referred to as ICI. Terminal immunostaining for Ki67, a marker of cell proliferation, was performed on both spheroids and organoids, demonstrating that MCF7 and ZR-75 organoids exhibited enhanced Ki67 expression across all donors compared to monoculture cancer spheroid culture. Clustering analysis identified distinct subpopulations with high (H), intermediate (I), and low (L) Ki67 expression within each dataset. Machine learning further revealed the direct effects of ASCs on Ki67 expression in addition to how ASC donor demographics may influence ASC-driven changes in the TME.

## Materials and methods

### Microfluidic device fabrication

The microfluidic droplet trapping array was fabricated using standard soft lithography and polydimethylsiloxane (PDMS) replication techniques previously described by Khan *et al.*^39^ Briefly, the device is composed of two polydimethylsiloxane (PDMS) layers, one layer containing a 100 μm tall microfluidic channel with a flow-focusing junction for droplet generation and another layer containing a 450-member array of circular traps that are 300 μm diameter and 300 μm deep. The device has two inlets, one for injection of Novec 7500 with a 0.5% m/v fluorosurfactant (008-fluorosurfactant, RAN Biotechnologies) and the other for the TA hydrogel precursors containing the cell suspension. Each device was fabricated from its own silicon master. Inlet and outlet holes were punched using a blunted 18-gauge needle before bonding the fluidic layer and trapping array devices together *via* treatment with oxygen plasma for 1 minute. Prior to experimentation, the device was coated with Aquapel for 2 min, followed by removal by applying high pressure nitrogen gas and then rinsed with Novec 7500 oil through the device. All tubing used to connect microfluidic devices and syringes were autoclaved to ensure sterility.

### Cell culture

MCF7 and ZR-75 cells (purchased from ATCC) were maintained with DMEM 1× (Corning) supplemented with 10% v/v HyClone cosmic calf serum (VWR Life Sciences Seradigm), 1% MEM essential amino acids (Quality Biological Inc.), 1% MEM non-essential amino acids (Quality Biological Inc.), 1 mM sodium pyruvate (Thermo Fisher Scientific), and 48 ng of insulin per mL (insulin, human recombinant dry powder, Sigma Aldrich). Cells were maintained in T-75 flasks in a humidified incubator at 37 °C and 5% v/v CO_2_. Cells were sub-cultured when they reached ∼80% confluency by first washing the cells with 1× phosphate-buffered saline (PBS: 137 mM NaCl, 10 mM Na_2_HPO_4_, 27 mM KCl, and 1.75 mM KH_2_PO_4_ at pH 7.4) containing 3.7 mM EDTA (Corning) and re-seeding into a new T-75 flask. Primary abdomen derived adipose stem cells (ASC) from female donors were purchased from LaCell. ASCs were used between passages 2 and 6 and maintained with MEM alpha/1× (Gibco) supplemented with 10% v/v fetal bovine serum – premium select (FBS, stem cell grade, Bio-Techne/R&D Systems, S11550), and 1% penicillin–streptomycin (VWR, 45000-616). ASCs were maintained in T-182.5 flasks in a humidified incubator at 37 °C and 5% v/v CO_2_. Cells were sub-cultured when they reached ∼80% confluency by first washing the cells with PBS and followed by treating the cells with 0.25% trypsin–EDTA (3 mL for one T-182.5 flask, VWR) for 3 min incubation at 37 °C before re-seeding into a new T-182.5 flask.

### Generation, isolation, and treatment of spheroids and organoids in the droplet microfluidic trapping array

**Fig. 1:**
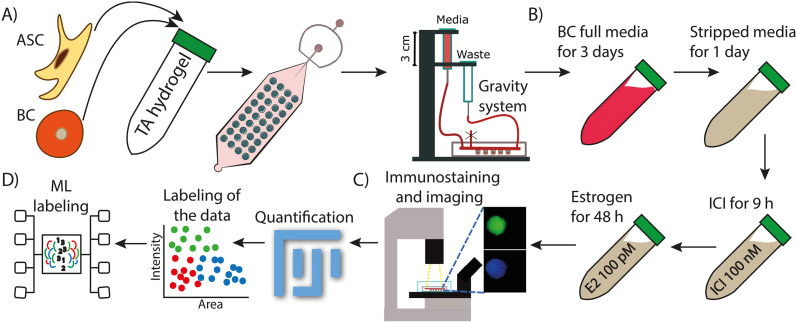
Experimental workflow of the droplet microfluidic approach for the 3D culture of spheroids and organoids. A) Suspending the organoids (or spheroids for monoculture) in the TA hydrogel scaffold followed by infusion into the device of BC cells and ASCs. Once formed, the spheroids were cultured for 7 days in a 3D printed scaffold to support continuous infusion and withdrawal of media. B) Four step culture approach to grow spheroids, remove exogenous stimuli, and treatment with ICI and/or estrogen to induce proliferation. C) Terminal immunostaining of spheroids for the proliferative marker Ki67. D) Image analysis, quantification, data clustering, and machine learning to identify different subpopulations based on fluorescence intensity and size.

The approach to produce single (spheroids) or co-culture (organoids) cell-laden droplets in the TA hydrogel scaffold using the microfluidic approach was previously described by Khan *et al.*^40^ Prior to experimentation, a 5 mL syringe containing only Novec 7500 was connected to the aqueous inlet to remove all the air from the device. The oil inlet was then connected to a 5 mL syringe containing Novec 7500 oil + fluorosurfactant using Tygon tubing. Following device preparation, the TA hydrogel/cell suspension was prepared. ASCs and/or BC cell lines (either MCF7 or ZR-75) were collected from the flask, suspended in media, mixed in a 1 : 1 ratio cancer cell to ASCs (in the case of the organoids), and then centrifuged at 400 rcf for 5 minutes. The supernatant was removed, and the cells were re-suspended in an 8.5 wt% TA hydrogel in extracellular buffer (ECB, pH 7.7) as previously described^39^ at a final density of 4 × 10^6^ cells per mL (Fig. 1A). The suspension was transferred into a 1 mL syringe which was connected to the device using a 23-gauge needle and Tygon tubing. Once the outlet tubing and the syringe with the surfactant were connected to the device, flow was initiated (oil with surfactant at a rate of 220 μL h^−1^ and cell-laden gel at a rate of 650 μL h^−1^) using two KD Scientific syringe pumps to generate cell-laden liquid hydrogel droplets at the flow focusing junction of the device until all traps were filled (∼1 minute). Afterwards, the device was cleaned by flowing oil at 2500 μL h^−1^ to remove non-trapped droplets. The oil/surfactant syringe was then swapped with a syringe containing only Novec 7500 oil which was flushed through the device for ∼35 min at a rate of 1000 μL h^−1^ to remove any residual fluorosurfactant from the device. Once that was done, a 3 mL syringe containing BC culture media supplemented with 1% v/v penicillin and streptomycin was connected to the hydrogel inlet to remove the oil at 1000 μL min^−1^. Once the device was filled with media and oil was evacuated, the device was coupled with a custom gravity driven flow media system to induce media and continuously feed the cells (Fig. 1A). The device was then placed in the CO_2_ incubator at 37 °C for the duration of the experiment to allow the spheroids and organoids grow. Growth media was replenished every 24 h. Brightfield images of the entire 450-member trapping array were collected daily to monitor spheroid formation and growth.

To evaluate endocrine therapy response in organoids/spheroids, aliquots of 100 μM fulvestrant (ICI 182780, Millipore Sigma, I4409-25MG) were made in dimethyl sulfoxide (DMSO, Millipore Sigma, D8418) and stored at −20 °C until they were used. Aliquots of 100 nM 17β-estradiol (E2, Millipore Sigma, E2758-1G) were made in DMSO and stored at −20 C. After culturing the organoids/spheroids for 3 days with full BC media, PBS was flowed for 30 minutes to remove any trace of media from the system. Next media free of exogenous estrogens (phenol free DMEM media containing 5% charcoal dextran FBS, 1% glutamax, 1% nonessential amino acid, 1% essential amino acid, 1% sodium pyruvate, and 1% penicillin–streptomycin) was flowed through the system for 24 h. Following this, to evaluate the effect of ICI in the organoids, 5% phenol free media with a final concentration of 100 nM ICI was flowed for 9 h followed by 48 h of continuous infusion of 5% phenol free media with 100 pM E2 (Fig. 1B). E2 media was replenished every 24 h for a total of 48 hours. To evaluate the effect of estrogen stimulation on the organoids, the same procedure was followed, except DMSO was used instead of ICI. A vehicle control experiment was also performed in the same conditions using DMSO instead of either ICI or E2.

### Immunostaining, fluorescent microscopy, and data acquisition

At the end of the 7 day drug response study, 4% v/v paraformaldehyde was flowed through the device using a syringe pump to fix the organoids at a rate of 7 μL min^−1^ for 2 h. The device was then washed with 1× PBS for 15 minutes at 15 μL min^−1^. The spheroids and organoids were permeabilized by flowing 0.5% v/v Triton X in PBS overnight at 7 μL min^−1^. A blocking step was performed next by flowing 1% v/v BSA in PBS for 1 h at 7 μL min^−1^. The antibody staining solution was prepared in sterile conditions by mixing 500 μL of 0.5% v/v BSA in PBS, 3 μL of nuclear stain Hoechst 33342 (60 μM), and 3 μL of anti-Ki-67 mouse monoclonal antibody (Alexa Fluor® 488, 3 : 500) or 3 μL of CD44 monoclonal antibody (IM7, Alexa Fluor® 488, 3 : 500). The staining solution was transferred to a 1 mL syringe and flowed through the device using a syringe pump at 1 μL min^−1^ for 7 h at room temperature in the dark. Lastly, the device was washed with 0.25% v/v BSA in PBS for 1 h at 7 μL min^−1^ before imaging. Fluorescent images were taken using a Leica DMi8 inverted microscope outfitted with a FITC filter cube (excitation 460–500 nm, emission 512–542 nm), DAPI filter (excitation 325–375 nm, emission 435–485 nm), and brightfield applications using a 20× objective. Fluorescent images were acquired using a Flash 4.0 high-speed camera (Hamamatsu) with a fixed exposure time of 900 ms for the FITC filter (green, Ki67) or 100 ms (green, CD44), 10 ms for DAPI (blue, nucleus), and 10 ms for brightfield (Fig. 1C). CD44, a known stem cell marker,^34^ was used to determine the area where the ASCs were located. Using FIJI, a threshold was set to identify where the CD44 signal was stronger, and that area was identified as composed by ASCs in the organoid. DAPI was used to identify the area of the entire spheroid or organoid. All fluorescent images were quantified using FIJI to analyze Ki67 expression. The threshold to identify the organoid was set using DAPI, and then both channels (FITC and DAPI) were quantified. The signal was normalized by subtracting the background to the mean signal of each channel and then dividing the FITC signal by the DAPI signal as shown in eqn (1).1
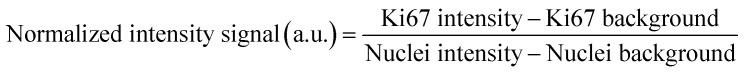


### Clustering and machine learning labeling

Data clustering and machine learning were performed for the large single spheroid and organoid data sets to identify unique subpopulations and how ASCs modulate endocrine response. All the data clustering analysis was performed using Python 3. The data clustering and machine learning process consisted of two steps: (i) data input and (ii) model selection and training. As part of the data input, the analysis of the spheroids data set proceed as follows: the normalized intensity signal (as calculated above) and area for all MCF7 or ZR-75 spheroids were clustered through an unsupervised learning process using the *K*-means algorithm to group the data based on the similarity of *K* values ranging from 2 to 6 to explore and visualize the data. Each cluster was then assigned a label ranging from 0 to *K*–1, where the higher the label, the greater the Ki67 normalized intensity. *K* = 3 was selected as the best grouping based on the experimentalist criteria and the creation of three meaningful subpopulations. A process of manually re-labeling some spheroids was then performed based on the treatment condition, normalized intensity signal, and experimentalist criteria. This was done to re-group some data points considering the conditions of the experiment and to make each group more cohesive. This process yielded three spheroid groups (for each BC cell line) with labels from 0 to 2 with 0 being the lowest Ki67 fluorescence intensity (L cluster) and 2 being the highest Ki67 fluorescence intensity (H cluster). Each experimental condition is composed of at least 300 data points.

As part of the model selection and training, the analysis was done using Python 3 to compare the spheroid and organoid data sets. The Gaussian Naïve Bayes classifier model was selected (an algorithm that assigns data points to predefined categories) to assess the impact of ASCs on organoid proliferation through supervised learning. The model was trained with 70% of the labeled monoculture data for either the MCF7 or ZR-75 spheroids. The algorithm was used to generate a predictive model, and the accuracy was evaluated with the remaining 30% of each BC spheroids data set. After the model was trained, optimized, and the accuracy was validated, it was used to classify unlabeled new data, namely the organoids. The normalized data from the organoids were introduced into the model and the output of this step was a labeled dataset indicating which cluster each organoid would be assigned based on the specified features (intensity and area). This method allowed for the categorization of organoids into the three pre-defined clusters, H, I, or L, based on Ki67 fluorescence intensity.

### Statistical analysis

All sets of experiments were performed as technical replicates and run in triplicates. Each experimental condition consisted of at least 300 data points. Statistical differences between groups were determined by standard one-way ANOVA and Tukey test using Origin software. *P*-Values <0.05 were considered significant (*), <0.01 very significant (***), or >0.05 non-significant (ns).

## Results and discussion

### 3D culture of ER^+^ breast cancer cells and ASCs as spheroids and organoids using a microfluidic droplet trapping array

**Fig. 2:**
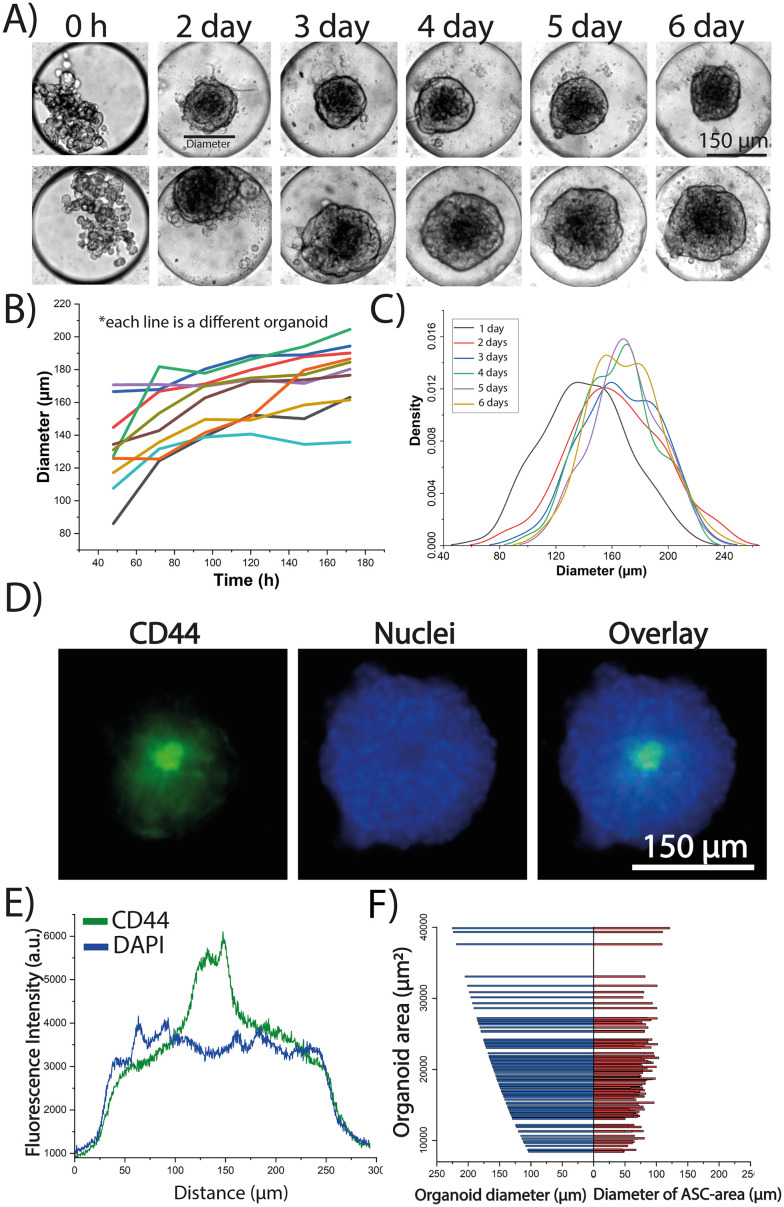
Time-dependent growth and spatial distribution of MCF7 and ASCs cells within the 3D organoids. A) Brightfield montage of the morphology and growth of two model MCF7 organoids cultured for 6 days in the device. B) Profile of the growth of nine model MCF7 organoids throughout 6 days of culture by measuring organoid diameter (each color is a different organoid). C) Diameter distribution of 100 model MCF7 organoids in days 1–6. D) Example fluorescent microscopy images of terminal CD44 and DAPI immunostaining of a model MCF7 organoid to show the spatial distribution of the ASCs. E) Line scan through the diameter of the MCF7 organoid in (D) showing the threshold used to identify the location of the ASCs and their area within the organoid (green line). F) Comparison between the diameter of the region of the organoid containing the ASCs (red) *vs.* the diameter of the entire organoid (blue). Both values are plotted against the total area of the organoid. Data is representative of 150 organoids.

Prior work by Khan *et al.* measured the diameter of MCF7 spheroids in the microfluidic device and found a gradual increase in spheroid size during a 7 day culture period that was correlated to cell growth.^39^ The TA hydrogel, previously characterized by Khan *et al.*,^40^ was used as a transient scaffold which degrades in ∼24–36 hours after cellular encapsulation. The gel provides a scaffold to help to create the 3D structure of the spheroids/organoids; however, after 72 hours the ECM in the spheroids/organoids is generated by the cells themselves. A similar approach was performed here to characterize the growth of monoculture ASC spheroids over a 6 day period. The spheroids transitioned from a dispersed aggregation of cells to a more compact structure (Fig. S1A†). After 24 h of culture, the spheroids exhibited a normal distribution with an average diameter of 169 ± 43 μm, and by day 4, the average diameter had decreased to 129 ± 13 μm (Fig. S1B†) indicating a trend toward cellular compaction. After six days in culture, it was found that the diameter remained relatively constant, though with a higher standard deviation (125 ± 46 μm) compared to day 4, suggesting variability in the spheroid morphology at this later time point. This supported by ∼50% of the spheroids at the day 6 time point beginning to spread, making it challenging to accurately measure their area (Fig. S1A,† row 3). Similar behavior has been reported by previous studies with Wolff *et al.* generating mesenchymal stem cells (MSC) spheroids in 3D culture and reporting a trend towards cell compaction for 28 days.^41^ Rovere *et al.* showed that spheroid size in MSCs influences senescence as they found that spheroids with diameters of ∼250 μm, after compaction, tend to keep a constant diameter after 72 h of culture.^42^ The growth of the organoids was next monitored for both MCF7 (Fig. 2A) and ZR-75 (Fig. S2A†) cell lines. The diameter of the organoids was tracked for six days of culture for 100 organoids in each case (MCF7 and ZR-75 with the same ASC donor). The change of the diameter of the ZR-75 organoids exhibited an average steeper slope (12.3 μm per day, Fig. S2B†) compared to the MCF7 organoids (4.9 μm per day, Fig. 2B) suggesting that ZR-75 organoids were growing faster than MCF7 organoids. The diameters in the MCF7 organoids showed a mostly normal distribution during the 6-day culture period with the maximum shifting to the right as the organoids grew (Fig. 2C). Conversely, the ZR-75 organoids started with a normal distribution but then changed with a right-shifted peak and left shifted tail suggesting the presence of a subset of smaller organoids growing slower than the rest (∼20%) (Fig. S2C†). The shift of the diameter distribution of the ZR-75 organoids to the right is more accentuated compared to the MCF7 organoids, which agrees with the differences of growth rate slopes. Overall, the growth profile of the organoids was similar to the profile of the BC spheroids in monoculture, where they tend to expand over time with no observed compaction.

The stem cell marker CD44 was used to identify the location and area of the ASCs to track their spatial distribution within the organoids. Terminal CD44 staining was first performed in the monocultured spheroids in all three cell lines to evaluate CD44 expression. Fluorescent microscopy images confirm a CD44 fluorescence intensity ∼5 times stronger in the ASCs (Fig. S3A†) compared to the MCF7 or ZR-75 cells alone (Fig. S3B†). This was further confirmed by performing a line scan across the spheroid that showed that CD44 levels in the ASCs were 5.2 ± 0.5 times higher when compared to MCF7 or ZR-75 spheroids (Fig. S3C and D†) confirming that CD44 expression can be used to track the spatial distribution of ASCs in the organoids. Terminal CD44 immunostaining of both MCF7 (Fig. 2D) and ZR-75 organoids (Fig. S2D†) found that the ASCs localized towards the center of the organoids when co-cultured with both BC cells lines. This is consistent with prior studies where aggregation patterns of heterotypic cells within the 3D architecture may have an influence in ECM distributions throughout the cellular assembly.^43,44^ This spatial distribution could be attributed to the fact that cell aggregation depends on the particular gene expression and phenotype and certain cells prefer to adhere to their own kind of cellular subunits.^45^ The area of the region of the organoid containing the ASCs was quantified by determining a threshold in the CD44 images to measure the ratio between the region occupied by the ASCs *versus* the entire organoid. A line scan across the organoid with the FITC image identified the ASC area (Fig. 2D and S2E,† green line) while the DAPI image identified the organoid area (Fig. 2D and S2E,† blue line). This threshold value was used for 150 organoids to demonstrate that the region the ASCs occupy within the organoid is independent of the size of the entire organoid size for both MCF7 and ZR-75 cells (Fig. 2F and S2F†). This suggests that when the organoid area increases, it is mostly due to the growth and expansion of the BC cells whereas the ASCs remain concentrated and compacted within the core. This is consistent with the monoculture ASC data in which the change of area of the spheroids was negligible after the spheroids form a compact mass at day 2 (Fig. S1†).^46^

### The presence of ASCs in the organoids drives Ki67 expression and endocrine response with differences across the donors

**Fig. 3:**
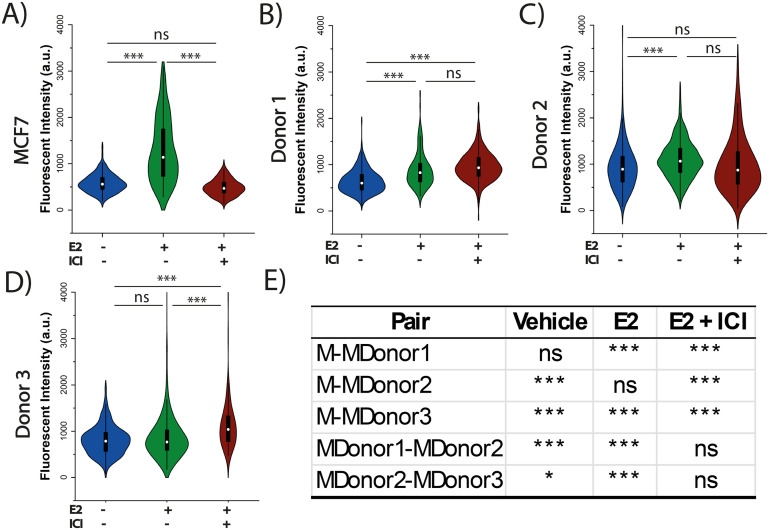
3D co-culture of MCF7 cells and ASCs show a difference in estrogen-mediated growth and endocrine response across different donors. A drug study was performed with quantification of terminal Ki67 expression under three culture conditions: (1) the vehicle control (blue plots), (2) exposure to 100 pM E2 for the final 48 h (green plot), or (3) exposure to 100 nM ICI for 9 h followed by 100 pM E2 for the final 48 h (brown plot). A) MCF7 spheroids. B) MCF7-ASC organoids from donor 1 age 37, BMI 36.3. C) MCF7-ASC organoids from donor 2, age 34, BMI 28.1. D) MCF7-ASC organoids from donor 3, age 68, BMI 28.3. E) Statistical analysis for vertical comparisons between MCF7 spheroids (M) and co-culture organoid (MDonor1–3). Table S1† expands on the nomenclature. *P*-Values <0.05 are considered significant (*), <0.01 are considered very significant (***), and >0.05 are considered non-significant (ns).

ASCs have been identified as a mediator of proliferation in the TME^47^ and a potential source of estrogen receptor activation.^20^ It is well established that patient characteristics can play a role in prognosis and tumor progression.^48,49^ Moreover, co-culture studies have demonstrated that ASCs induce heterogeneity in ER^+^ BC proliferation and ER activation.^20,21,50^ Here, ASCs derived from three different female donors with differences in age and BMI were used to investigate how donor characteristics play a role in inter-tumor heterogeneity driving endocrine response. As shown in Table S1,† two of the donors are young (<40) while one is aged (>65) and two of the donors are considered overweight (BMI 25–30) while one donor was classified as class II obese (BMI >35). To evaluate the effect of ASCs on ER^+^ BC, both MCF7 and ZR-75 spheroids and organoids were interrogated under three different conditions: 1) a vehicle control to determine basal levels of Ki67 expression in the absence of ER regulation, 2) exposure to 100 pM E2 for 48 h to measure Ki67 expression following E2 stimulation, or 3) exposure to 100 nM ICI for 9 h followed 48 h of 100 pM E2 to determine how the spheroids respond to the inhibitory effect of ICI. Ki67 expression was used as a proliferation marker to evaluate the response to the treatment based on literature and our prior studies.^39^ Both monocultured MCF7 and ZR-75 spheroids exhibited enhanced Ki67 expression following E2 treatment alone and diminished E2 mediated Ki67 expression following the pretreatment of ICI, where ICI treated spheroids were similar to the vehicle control (Fig. 3A and S4A,†Table 1). The average value of the normalized Ki67 intensity in the MCF7 and ZR-75 E2 + ICI exposed spheroids was 488 ± 14 and 417 ± 29, and the vehicle control treatment was 578 ± 14 and 468 ± 31, respectively. E2 exposed spheroids had elevated Ki67 expression of 1298 ± 66 and 1044 ± 56 for MCF7 and ZR-75 respectively, suggesting that the spheroids respond to the treatment conditions. Monocultured ASCs spheroids exhibited increased Ki67 expression in both E2 (1736 ± 82) and E2+ ICI (1501 ± 50) treatments, compared to vehicle control (869 ± 36) as shown in Fig. S5.† While ICI acts as an ER degrader, the impact of ICI in ASCs may be explained through expression of alternate ER receptors in ASCs, such as the G protein-coupled estrogen receptor (GPER).^47^ E2 is able to stimulate GPER however ICI has a reported lower affinity towards this receptor.^48^

**Table 1:** Variations in Ki67 expression in the spheroids and organoids following endocrine treatment when compared to their vehicle control

Compared to vehicle	MCF7				ZR-75			
	Mono-culture	Donor 1	Donor 2	Donor 3	Mono-culture	Donor 1	Donor 2	Donor 3
E2	Elevated	Elevated	Elevated	No change	Elevated	No change	No change	Elevated
ICI + E2	No change	Elevated	No change	Elevated	No change	Repressed	Repressed	Elevated

A statistical comparison was performed to evaluate Ki67 expression levels for each of the donor organoids compared to the spheroids to allow for an evaluation of the differences across treatment conditions in the presence or absence of ASCs in the tumor. A statistical comparison between MCF7 monocultured spheroids and co-cultured organoids showed significant differences in (1) basal Ki67 expression in donors 2 and 3, (2) E2 treatment in donors 1 and 3, and (3) E2 + ICI treatment in all three donors (Fig. 3E). A similar comparison in ZR-75 spheroids and organoids showed significant difference in (1) basal Ki67 expression in donors 1 and 2, (2) E2 treatment in donor 1, and (3) E2 + ICI treatment in donors 2 and 3 (Fig. S4E†). Comparison across vehicle control treated groups (BC spheroid *vs.* BC organoid), where the presence of ASCs is evaluated without the effect of any treatment as an external factor, has shown similar results in previous studies in which the presence of young ASCs drive proliferation in ER^+^ cells.^21,32^ The organoids exposed to ICI are of special interest since they can provide insight on how ASCs can modulate the endocrine response of the tumor when, in normal conditions after neo-adjuvant treatment, the proliferation levels should be at the basal level to indicate a favorable response to therapy. With the exception of donor 1 in the ZR-75 organoids, all donors with both BC cell lines presented higher levels of Ki67 expression when compared to the monocultured spheroids (Fig. 3E and S4E†). A comparison between MCF7 organoids across the three donors showed significant differences: donor 2 induced higher Ki67 expression when compared to donor 1 and 3 in the vehicle and E2 treatments, but no change in the E2 + ICI treatment (Fig. 3E) further supporting a role for ASCs in endocrine resistance. This was not the case in the ZR-75 organoids which showed significant differences in Ki67 expression across all three donors under all three treatment conditions. Donor 2 induced higher Ki67 expression than 1 and 3 in the vehicle treatment, while in the E2 and ICI + E2 exposed organoids, donor 2 had higher Ki67 compared to donor 1, but donor 3 presented higher Ki67 than donor 2.

Treatments in the co-culture organoids demonstrated unique responses based on both breast cancer cell line and ASC donor. Table 1 shows the variations in Ki67 response of the organoids after endocrine treatment when they are compared to their own vehicle control. Under E2 exposure, MCF7 organoids with donors 1 (37 years, BMI 36.3, Fig. 3B) and 2 (34 years, BMI 28.6, Fig. 3C), and ZR-75 organoids with donor 3 (68 years, BMI 28.3, Fig. S4D†) showed an increase in Ki67 expression, while MCF7 organoids with donor 3, and ZR-75 organoids with donor 1 and 2 showed no changes. Under ICI + E2 exposure, donor 3 with both cancer cell lines (Fig. 3D and S4D†), and donor 1 with MCF7 cells (Fig. 3B) showed an increase in Ki67 expression compared to vehicle control, suggesting that cells in co-culture can respond to E2 even in the presence of a SERD. Under the same treatment (ICI + E2), donor 2 with MCF7 cells did not show significant change in Ki67 levels, while donors 1 and 2 with ZR-75 cells showed repressed Ki67. This result suggests that in this case, ASCs did not induce a significant effect under ICI treatment. Interestingly, when comparing Ki67 levels between E2 and ICI + E2, MCF7 organoids maintained the same levels between both treatments in donor 1 and 2, while donor 3 even increased its Ki67 in the ICI + E2 when compared to the E2 treatment. All donors in ZR-75 organoids showed a decreased Ki67 in the ICI + E2 treatment compared to the E2 exposed organoids, indicating that even though ASCs in donor 3 were able to enhance proliferation under ICI + E2, those organoids still expressed less Ki67 than the E2 exposed ones. Results of this study suggest that MCF7 organoids provide some degree of endocrine resistance, but not in the ZR-75 organoids. These findings support the role for ASC mediated inter-tumor heterogeneity and tumor progression in BC endocrine response, as it has been established that the secretome of ASCs strongly influences breast cancer behavior.^51^

### Data clustering of 3D spheroids identified distinct subpopulations that drive intra-tumor heterogeneity

**Fig. 4:**
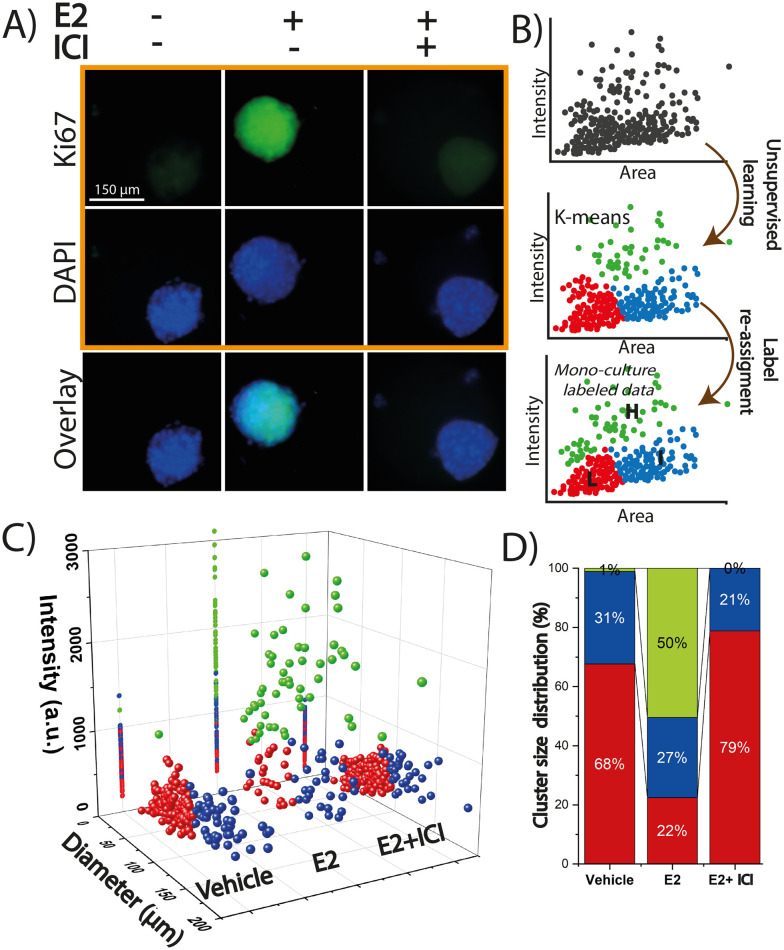
Data clustering of monocultured MCF7 spheroids to label the data to identify distinct subpopulations. A) Representative fluorescence microscopy images of MCF7 spheroids immunostained for Ki67 expression under the three treatment conditions. B) Schematic representation of clustering based on normalized fluorescence intensity and area to label the data into three groups of high (H, green), intermediate (I, blue), or low (L, red) Ki67 expression. C) Visualization of all the monoculture MCF7 spheroids labeled into the three groups and separated across all conditions. D) Population distribution of the MCF7 spheroids in each label for each condition. The percentage is the fraction of organoids in each cluster per treatment divided by the total amount of organoids per treatment.

Despite the initial response of luminal A and B breast cancer subtype to endocrine therapies, many BC cells have *de novo* or develop resistance. One of the reasons why this occurs is the existence of subpopulations of BC cells within the tumor with differences in tumorigenicity, metastatic potential and drug sensitivity.^5,6^ Machine learning algorithms can uncover patterns and relationships that might not be readily apparent through conventional analysis by analyzing large, complex datasets from mono and co-culture experiments. To build upon the above analysis, machine learning techniques were used to evaluate the effects of ASCs on tumor proliferation in the 3D organoid models with a focus on understanding how donor-specific factors, such as age and BMI, influence this interaction. The entire data set of the spheroids (monocultured MCF7 or ZR-75) for all three treatments was first stratified based on spheroid area and normalized Ki67 fluorescence intensity through unsupervised learning to create three labels for groups with high (H), medium (I), and low (L) Ki67 expression (Fig. 4A and B) with the naming scheme for the clusters shown in Table S2.† For example, MCF^L^ is the subpopulation of MCF7 spheroids in the cluster low (L) range of Ki67 fluorescence intensity. When groups were defined, 99% of the data points of the MCF7^H^ group (which has the highest Ki67 expression in MCF7 spheroids) were found in the E2 treatment (Fig. 4C, green dots). The MCF7^L^ was characterized as having the lowest Ki67 expression and smallest area (Fig. 4B, red dots), while the MCF7^I^ group exhibited a slightly higher Ki67 expression and larger spheroid area (Fig. 4B, blue dots). The same trend was observed for ZR-75 spheroid groups. The population distributions were then calculated for the labeled MCF7 (Fig. 4D) and ZR-75 spheroids (Fig. S6B†) for all three treatment conditions to quantify the percentage of spheroids in each of the three groups or clusters. This percentage represents the fraction of spheroids/organoids (or data points) in each cluster per treatment divided by the total amount of spheroids/organoids (or data points) per treatment. Most of the MCF7 and ZR-75 spheroids occupied the I and L labels (red and blue) in the vehicle and E2 + ICI treatments with the largest percentage in the L label for both cell types. This suggests only a small number (∼1%) of MCF7 spheroids exhibit high basal levels of Ki67 expression. The percentage of ZR-75 spheroids that fell in the H label under basal and E2 + ICI treatment was slightly higher when compared to MCF7 spheroids (9% and 4% respectively); suggesting that there are more spheroids that are less susceptible to SERD-induced suppression of Ki67 expression in ZR-75 cells. However, the general trend for the vehicle and ICI + E2 treatments for both BC spheroids was the same: most of the spheroids fell in the L group (Fig. S6†). As expected, most of the MCF7 and ZR-75 spheroids reside in the H label during E2 treatment confirming that both ER^+^ cell lines are highly responsive to E2 induced Ki67 expression. These findings highlight intra-tumor heterogeneity in the BC cell lines with select groups of cells being responsive or non-responsive to ET. The average intensity and spheroid diameter per cluster was noted to be different between MCF7 and ZR-75 spheroids, whose values are listed in Table S3.† When measuring the correlation (how strong the dependance is) between the variables (Ki67 intensity and area of the spheroid) for the whole data set (either MCF7 or ZR-75 spheroids), the MCF7 spheroids exhibited a weak dependency (or correlation) of 0.35, suggesting that Ki67 intensity is not affected by the size of the spheroid. The ZR-75 spheroids presented a correlation of 0.68 indicating that ZR-75 spheroids have a stronger dependency between these variables suggesting that the size of the spheroid may enhance Ki67 expression. As seen in Fig. S6A,† there is a trend for the data points to have higher Ki67 intensity as the diameter increases. A study has previously shown that the profile of Ki67 expression inside Capan-2 pancreatic cancer spheroids decreases from the edge to the core of the spheroid with the authors claiming that the proliferative zone is restricted to the outer 150 μm layer.^52^ The spheroids in this study were smaller than 250 μm in diameter, which suggests that the Ki67 profile across the spheroid does not exhibit relevant differences. Data clustering was then performed on monocultured ASC spheroids from donor 3 which found that they exhibited some difference in their population distribution when compared to the MCF7 and ZR-75 spheroids (Fig. S6C and D†). Contrary to the BC spheroids, the E2 + ICI exposed ASC spheroids presented similar Ki67 levels to those exposed to E2 (as shown in Fig. S5†), which caused higher subpopulations (18% and 41%) of ASC spheroids in the H (green) cluster in the E2 + ICI and E2 treatments respectively. This could be attributed to the expression of the GPER in the ASCs and lower affinity of the ICI to this estrogen receptor leading to no significant growth inhibition. As for the vehicle control, only a small fraction (∼1%) of ASC spheroids showed high Ki67 expression being most of the spheroids grouped in the L and I cluster similar to the BC spheroids. The ASC spheroids also showed a higher basal Ki67 expression in the absence of hormones compared to the BC spheroids (869 ± 36 *vs.* 578 ± 14 for the MCF7 and 468 ± 31 in the ZR-75) with both clusters, ASC^L^ and ASC^I^, exhibiting significantly higher Ki67 expression levels than MCF7^L^, MCF^I^, ZR-75^L^, and ZR-75^I^ (Table S3†).

### BMI plays a role in endocrine response in the MCF7 organoids while age is a more predominant factor in the ZR-75 organoids

**Fig. 5:**
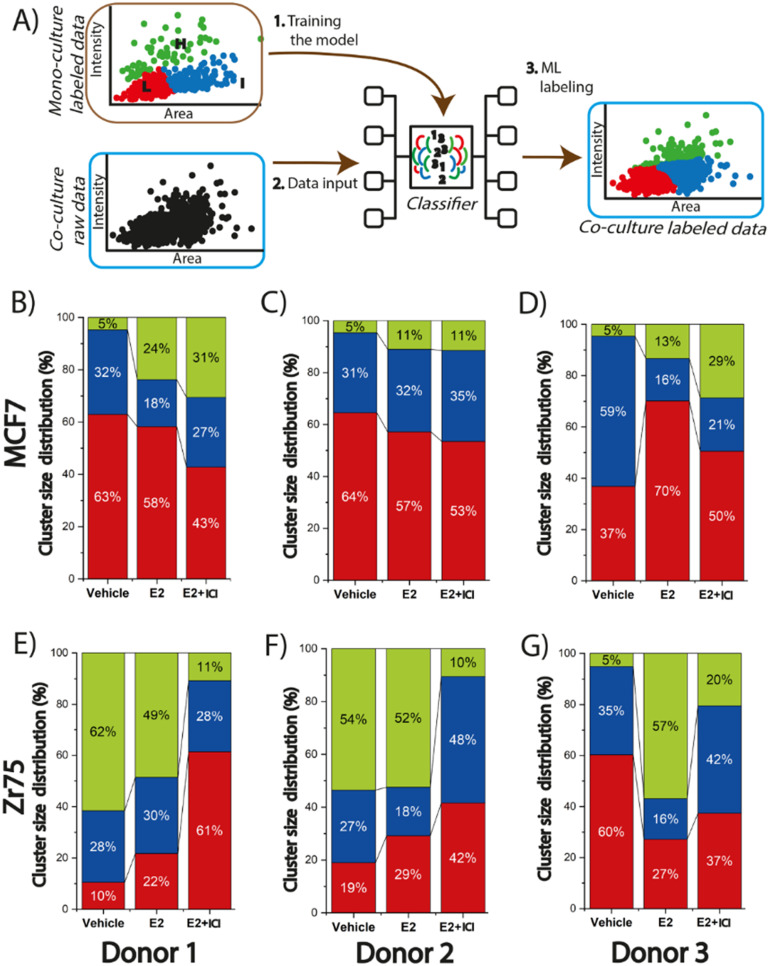
Organoid labeling through machine learning identifies subpopulations of ASC enhance proliferative marker expression across different treatments in a donor specific manner. A) Schematic representation showing how the predictive model was trained to get the unlabeled co-culture data and assign labels. The pre-labeled spheroids were introduced in the model to train it, then the unlabeled organoid data set was evaluated by the model to assign labels based on the predefined groups. Population distribution of the organoids for the H, I, and L labels in MCF7 cells co-cultured with donor 1 (B), donor 2 (C), and donor 3 (D). Population distribution of the organoids for the H, I, and L labels in ZR-75 cells co-cultured with donor 1 (E), donor 2 (F), and donor 3 (G).

The defined and labeled spheroid groups for the MCF7 and ZR-75 spheroids provide a foundation for examining different levels of Ki67 expression across different treatments in the BC-ASC organoids. Machine learning was then used to train a predictive model to label the organoids data for all three donors in H, I, and L groups (Fig. 5A). The accuracy of the trained model was 99% for MCF7 spheroids and 91% accuracy for ZR-75 spheroids. By applying this approach, it was possible to assess how the presence of ASCs modulates organoid Ki67 expression levels both in the presence and absence of endocrine therapy. Visualization of all the labeled organoids across all three donors and treatment conditions into the three subpopulations shows higher percentages of H labeled organoids, especially in the vehicle and E2 + ICI treatments, when compared to the monocultured spheroids (Fig. S7†). There was a greater percentage of cells (5%) in the H label in the vehicle control across all three donors in the MCF7 organoids compared to the 1% in the spheroids (Fig. 5B–D). The MCF7 organoids also exhibited a much larger percentage (11–31%) in the H cluster in E2 + ICI treatment when compared to the 0% in the spheroids (Fig. 5B–D). This trend varied across donors with H percentages of 31%, 11%, and 29% for donors 1–3 respectively. This suggests that ASCs provide the MCF7 cells with some degree of endocrine resistance resulting in enhanced expression of Ki67 in a subpopulation of cells and that this response is linked to donor attributes. ASCs from donor 2 (young, low BMI) presented a less pronounced effect than donors 1 (young, high BMI) and 3 (old, high BMI). The contrast in response between donors 1 and 2 in the MCF7 organoids suggest that donor characteristics, such as BMI, could be a driving factor in endocrine response. The organoids from donor 3, with a similar BMI to donor 2, exhibited enhanced Ki67 levels, suggesting that age may also play a role driving endocrine response. Previous studies have shown that obesity plays a role in BC since it has been linked with poor prognosis, increased risk of recurrence, and resistance to therapies for both pre- and postmenopausal women.^53,54^ It has been suggested that one of the reasons why this may happen is because BC cells have the ability to stimulate ASCs to secrete cytokines.^51^ For example, obese ASC donors (BMI > 30) were found to have elevated leptin secretion and promote growth and metastasis when compared to ASCs from lean donors (BMI < 25) in BC.^55,56^ Interestingly, in the MCF7 organoids in the vehicle treatment, ASCs induce proliferation similarly and independently of donor characteristics; however, when it to comes to endocrine response after the exposure to ICI, elevated BMI may play a more prominent role. This finding can be correlated with the study by Ling *et al.*, where they demonstrated that ASCs derived from obese mice co-cultured with MCF10AT1, show a higher ability to induce tumor cell migration in collagen fibers.^57^ While prior works support ASC elevated donor BMI as a mechanism of endocrine resistance, the current study is limited in adequate donor pools to fully demonstrate this phenomena. Future microfluidic co-culture studies should focus on the impact of ASC donor demographics such as BMI and age on the breast cancer endocrine response.

The presence of the ASCs showed a more prominent effect in the ZR-75 organoids, when compared to the MCF7 organoids with significantly enhanced Ki67 expression across all three donors in the presence and absence of E2. In the presence of ICI, the H cluster presented a larger fraction of the organoids compared to the spheroids that varied across donors. Initially 4% of the ZR-75 spheroids resided in H cluster after ICI exposure; however, this increased to 11%, 10%, and 20% for donors 1–3 respectively (Fig. 5E–G). Organoids from donor 3 yielded a larger fraction in the H cluster when compared to donors 1 and 2. This similarity between donors 1 and 2 suggests a role for age over BMI in driving endocrine resistance in the ZR-75 organoids. In the absence of E2, ASCs from donors 1 and 2 drive a substantial proportion (more than 50%) of the organoids into the H cluster, potentially due to a higher release of growth factors.^21^ This result supports a role for ASC donor age in driving enhanced expression of proliferative markers, but potentially through a different mechanism from the organoids exposed to ICI. A similar study found that ASCs from younger donors enhanced proliferation and phosphorylation of ERα S167 in ER^+^ BC cells when compared to ASCs from older donors but both, proliferation and phosphorylation of ERα were knocked down after the application of tamoxifen,^21^ supporting the role of young ASCs in enhancing proliferation in the absence of ER inhibitors. When ZR-75 organoids were exposed to ICI, ASCs from the older donor (donor 3) presented higher Ki67 expression in the organoids. This result suggests that there are differences in the susceptibility to the SERD between ASCs from young *vs.* old donors. The vehicle control treatment in the ZR-75 organoids of donors 1 and 2 exhibit higher Ki67 expression levels compared to the ZR-75 spheroids (Fig. S4D†). The labeling of the organoids shows that this difference is driven by more of the 50% of the data points in the organoids that are statistically higher (being part of the H cluster) than the Ki67 expression levels in the ZR-75 spheroids. These results suggest that the capacity of ASCs to modulate the TME varies depending on donor characteristics, specifically age, in the case of the ZR-75 organoids and BMI in the case of the MCF7 organoids. In the absence of treatment, ASCs from younger donors appear to drive greater tumor proliferation, while ASCs from older donors seem to be able to better sustain tumor proliferation in the presence of ICI, indicating a potential age-dependent difference in ASC-mediated tumor responses. Conversely, ASCs from donor with a high BMI (donor 1) seems to enhance endocrine resistance after the application of ICI. Regardless of cell type, ASCs from all donors were found to enhance Ki67 expression in the organoids.

## Conclusions

This study used a microfluidic platform integrated with a TA hydrogel to perform 3D co-culture of primary adipose-derived stem cells (ASCs) from donors with varying age and BMI with two model ER^+^ BC cell lines. The purpose was to investigate how ASC co-culture influences the tumor response to ET, specifically ICI, using data clustering and machine learning techniques. The results revealed that, when co-cultured, ASCs aggregated at the core of the organoid while the BC cells would continue to expand radially. ASC-mediated heterogeneity was found to play a significant role in modulating the BC response to ET in the 3D organoids. Notably, there was a variability in the effects of ASCs between the two different ER^+^ BC cell lines. In MCF7 organoids from a donor with a higher BMI, the presence of the ASCs better promoted sustained and higher Ki67 expression even after ICI exposure, while in both organoids, donor age was demonstrated to play a role with ASCs enhancing Ki67 expression after ICI exposure. In the absence of E2, it was found that in the ZR-75 organoids, ASCs from young donors substantially increased Ki67 expression. The study provided initial findings supporting that donor attributes can modulate the endocrine response in ER^+^ BC with future studies aimed to expand upon these findings to interrogate additional donors to gain a greater understanding and better characterize age, BMI, and ASCs presence impact tumor response to endocrine therapy.

## Author contributions

B. O. designed the experiment, fabricated and characterized the wafers and micro-devices, performed all the experiments, quantified part of the data, ran all the analysis, and wrote the manuscript. C. C. analyzed part of the data. E. M. and A. M. supervised the research and reviewed the manuscript.

## Conflicts of interest

The authors declare no competing interests.

## Supplementary Material

LC-025-D5LC00320B-s001

## Data Availability

All raw and processed data presented in this manuscript has been uploaded and stored to the Clemson University OneDrive where it is securely maintained under the direction of the corresponding author. Back-ups of all the data are also maintained on external hard drives located in the office of the corresponding author. All data, raw and processed, can and will be made available to any interested parties. They are encouraged to directly contact the corresponding author (Dr. Adam Melvin, melvina@clemson.edu) to request access to the data.
