# Supplementary material for: Droplet microfluidics integrated with machine learning reveals how adipose-derived stem cells modulate endocrine response and tumor heterogeneity in ER+ breast cancer
Source: Lab Chip. 2025 Jun 13;25(15):3817–30. doi: 10.1039/d5lc00320b (PMC12177710; doi:10.1039/d5lc00320b)
Supplement: LC-025-D5LC00320B-s001 [file LC-025-D5LC00320B-s001.pdf]

Supplementary Information (SI) for Lab on a Chip.
This journal is © The Royal Society of Chemistry 2025

Supplemental Material

Droplet microfluidics integrated with machine learning reveals how adipose-derived stem cells modulate endocrine response and tumor heterogeneity in ER^+ breast cancer

Braulio Andres Ortega Quesada,[1] Calley Chauvin,[2] Elizabeth Martin, [3*] Adam Melvin[1*]

[1]*Department of Chemical and Biomolecular Engineering, Clemson University, Clemson, SC 29634*

[2]*Department of Biological and Agricultural Engineering, Louisiana State University, Baton Rouge, LA,70803*

[3]*Department Medicine, Section Hematology and Medical Oncology, Tulane University, New Orleans, LA, 70118*

**Corresponding author*

Contents

Figure S1. Time-dependent growth of ASCs as 3D spheroids…………………………………………..3

Figure S2. Time-dependent growth and spatial distribution of ZR-75 and ASCs cells within the 3D organoids..4

Figure S3. CD44 expression profile is enhanced in ASC spheroids when compared to MCF7 and ZR-75 spheroids. 5

Figure S4. 3D co-culture of ZR-75 cells and ASCs show a difference in estrogen-mediated growth and endocrine response across different donors 6

Figure S5. Distribution of Ki67 expression in the ASC 3D spheroids after drug treatment. 7

Figure S6. Data clustering of monocultured ZR-75 and ASC spheroids to label the data to identify distinct subpopulations 8

Figure S7. Organoids are labeled into three groups based on fluorescence intensity and area using supervised machine learning...9

Table S1. ASC donor characteristics 10

Table S2. Nomenclature system for each cluster...11

Table S3. Average intensity and diameter per cluster in the organoids and spheroids. This table shows the average Ki67 fluorescence intensity and diameter in each of the clusters. 12

Table S4. Intensity and diameter per cluster separated by condition in the MCF7 organoids. This table shows the average Ki67 fluorescence intensity and diameter in each of the clusters separated by treatment condition. 13

Table S5. Intensity and diameter per cluster separated by condition in ZR-75 organoids. This table shows the average Ki67 fluorescence intensity and diameter in each of the clusters separated by treatment condition. 14

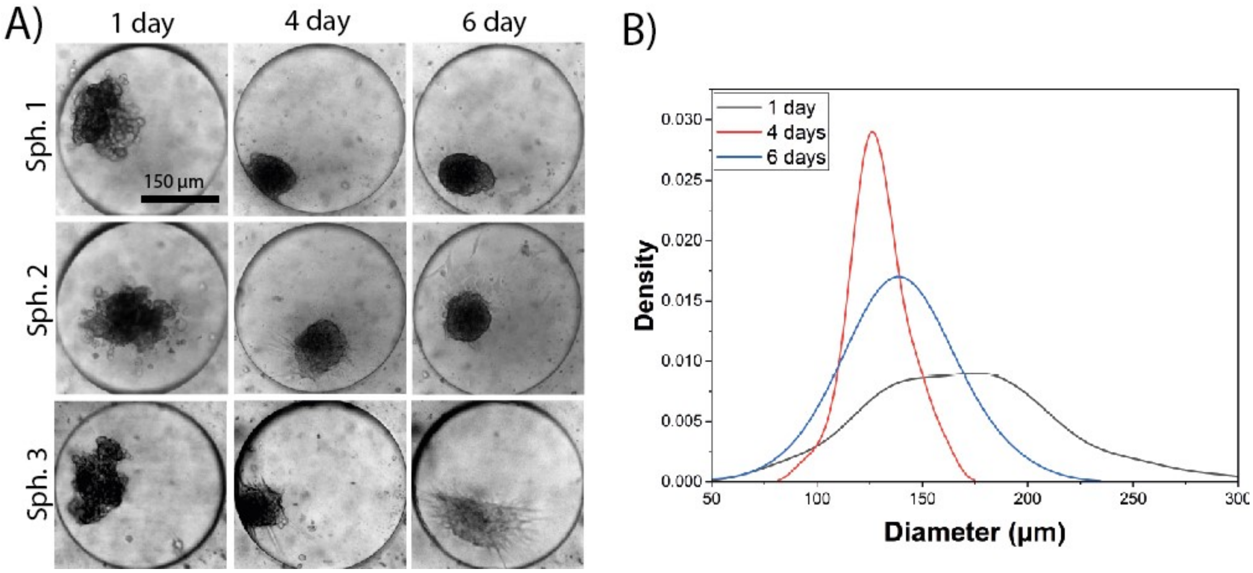

Figure S1. Time-dependent growth of ASCs as 3D spheroids. *A) Brightfield montage of the morphology of three model single culture ASC spheroids from donor 3 cultured for 6 days in the microfluidic device. B) Diameter distribution of 75 model ASC spheroids at 1, 4, and 6 days.*

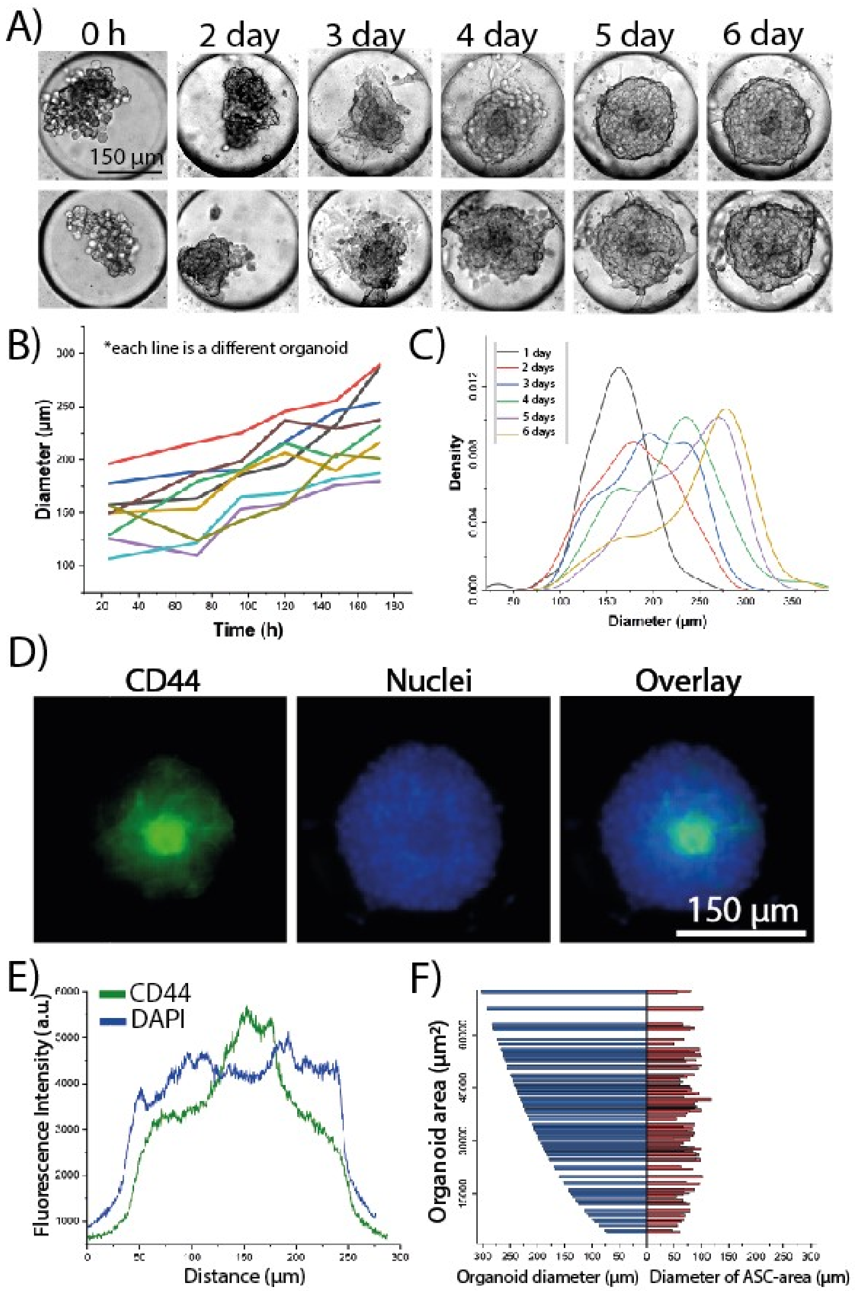

Figure S2. Time-dependent growth and spatial distribution of ZR-75 and ASCs cells within the 3D organoids. *A) Brightfield montage of the morphology and growth of two model ZR-75 organoids cultured for 6 days in the device. B) Profile of the growth of nine model ZR-75 organoids throughout 6 days of culture by measuring organoid diameter (each color is a different organoid). C) Diameter distribution of 100 model ZR-75 organoids at days 1- 6. D) Example fluorescent microscopy images of terminal CD44 and DAPI immunostaining of a model ZR-75 organoid to show the spatial distribution of the ASCs. E) Line scan through the diameter of the ZR-75 organoid in (D) showing the threshold used to identify the location of the ASCs and their area within the organoid (green line). F) Comparison between the diameter of the region of the organoid containing the ASCs (red) vs. the diameter of the entire organoid (blue). Both values are plotted against the total area of the organoid. Data is representative of 150 organoids.*

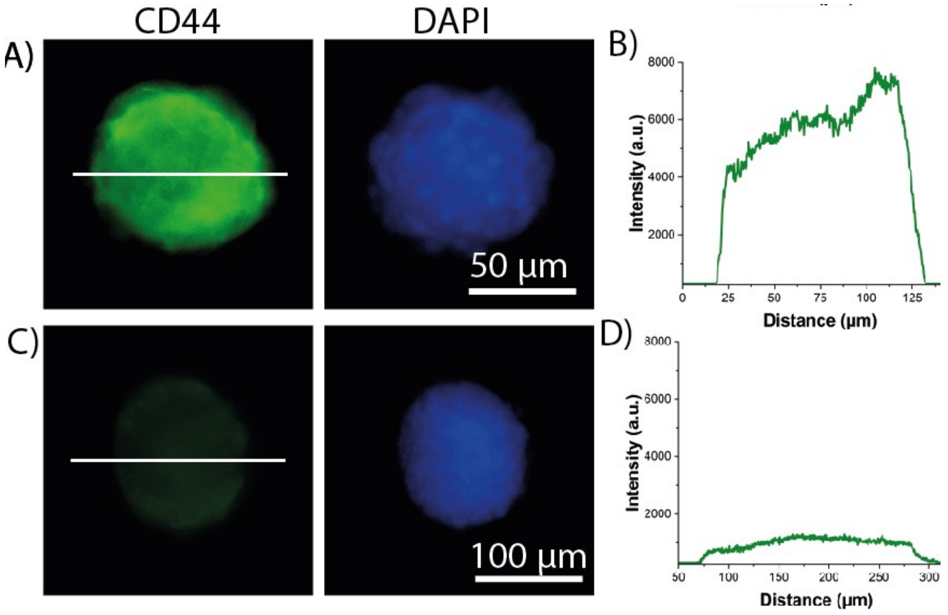

Figure S3. CD44 expression profile is enhanced in ASC spheroids when compared to MCF7 and ZR-75 spheroids. *A) Model ASC spheroid stained for CD44 (green) and nuclei (blue) showing the CD44 profile with a line scan. B) Quantification of fluorescence intensity of CD44 through the line scan in the ASC spheroid. C) MCF7 spheroid stained for CD44 (green) and nuclei (blue) showing the CD44 profile with a line scan. D) Quantification of fluorescence intensity CD44 through the line scan in the MCF7 spheroid. Data here is representative of 100 spheroids per each case.*

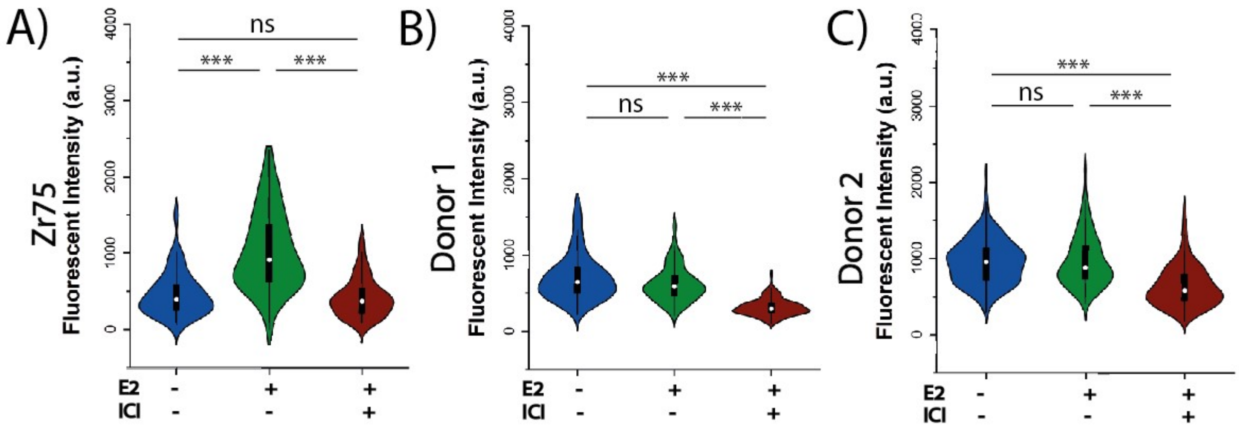

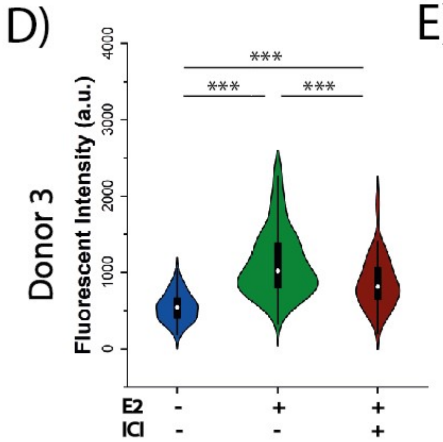

E)

Pair	Vehicle	E2	E2 + ICI
Z-ZDonor1	***	***	ns
Z-ZDonor2	***	ns	***
Z-ZDonor3	ns	ns	***
ZDonor1-ZDonor2	***	***	***
ZDonor2-ZDonor3	***	***	***

Figure S4. 3D co-culture of ZR-75 cells and ASCs show a difference in estrogen-mediated growth and endocrine response across different donors*. A drug study was performed with quantification of terminal Ki67 expression under three culture conditions: (1) the vehicle control (blue plots), (2) exposure to 100 pM E2 for the final 48 h (green plot), or (3) exposure to 100 nM ICI for 9 h followed by 100 pM E2 for the final 48 h (brown plot). A) ZR-75 spheroids. B) ZR-75-ASC organoids from Donor 1 age 37, BMI 36.3. C) ZR-75-ASC organoids from Donor 2, age 34, BMI 28.1. D) ZR-75-ASC organoids from Donor 3, age 68, BMI 28.3. E) Statistical analysis for vertical comparisons between ZR-75 spheroids (Z) and co-culture organoid (ZDonor1-3). Table S1 expands on the nomenclature. P-values <0.05 are considered significant (*), <0.01 are considered very significant (***), and >0.05 are considered non-significant (ns).*

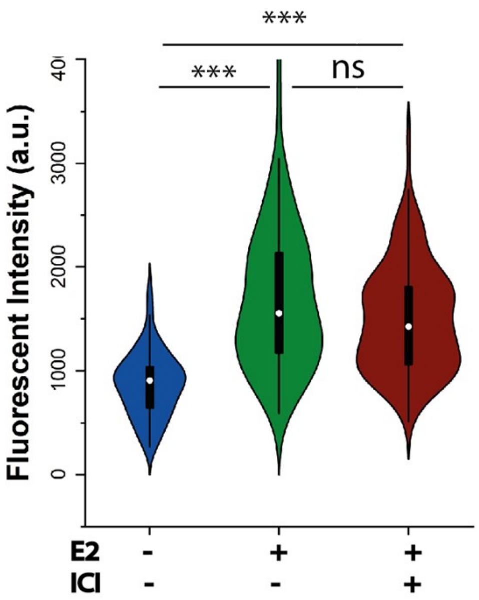

Figure S5. Distribution of Ki67 expression in the ASC 3D spheroids after drug treatment. *A drug study was performed on ASC spheroids derived from donor 3 with quantification of terminal Ki67 expression performed under three culture conditions: (1) the vehicle control (blue plot), (2) exposure to 100 pM E2 for the final 48 h (green plot), or (3) exposure to 100 nM ICI for 9 h followed by 100 pM E2 for the final 48 h (brown plot).*

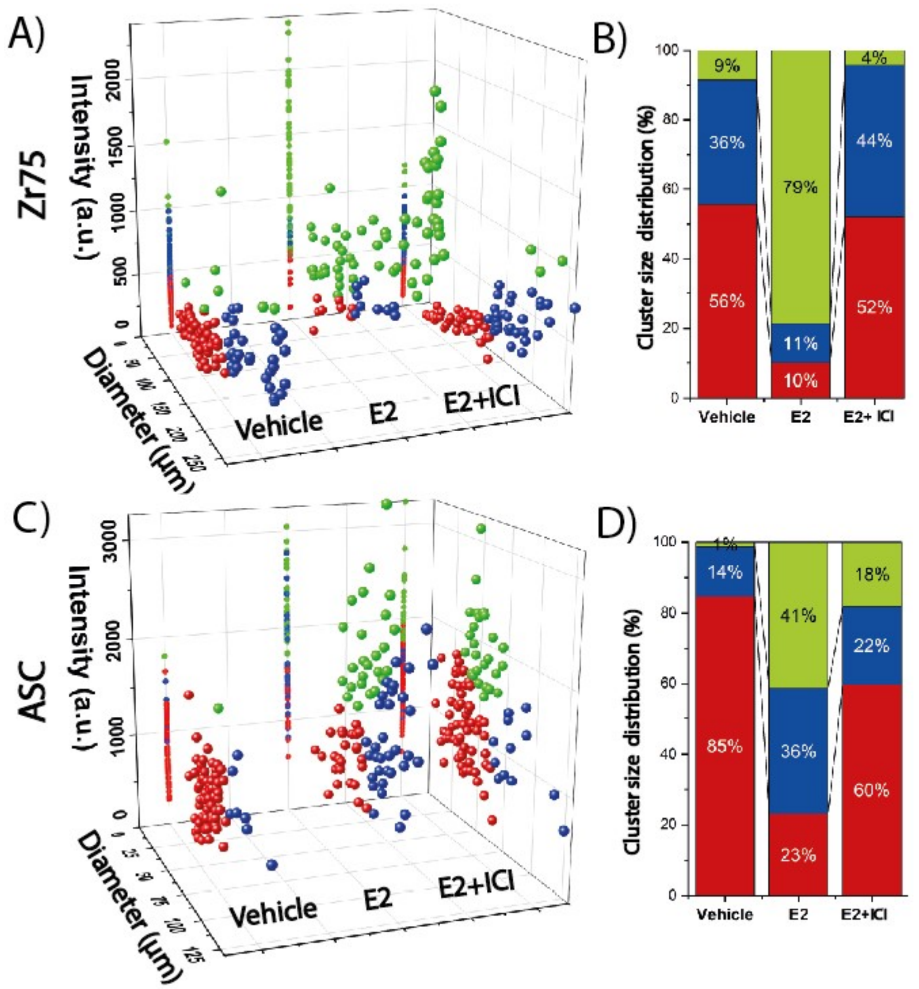

***Figure S6. Data clustering of monocultured ZR-75 and ASC spheroids to label the data to identify distinct subpopulations**. A) Visualization of all the monoculture ZR-75 spheroids labeled into the three groups and separated across all conditions. B) Population distribution of the ZR-75 spheroids in each label for each condition. C) Visualization of all the monoculture ASC spheroids labeled into the three groups and separated across all conditions. D) Population distribution of the ASC spheroids in each label for each condition. The percentage is obtained by dividing the number of data points in each cluster per treatment by the total amount of data points in the treatment (green cluster H, blue cluster I, and red cluster L).*

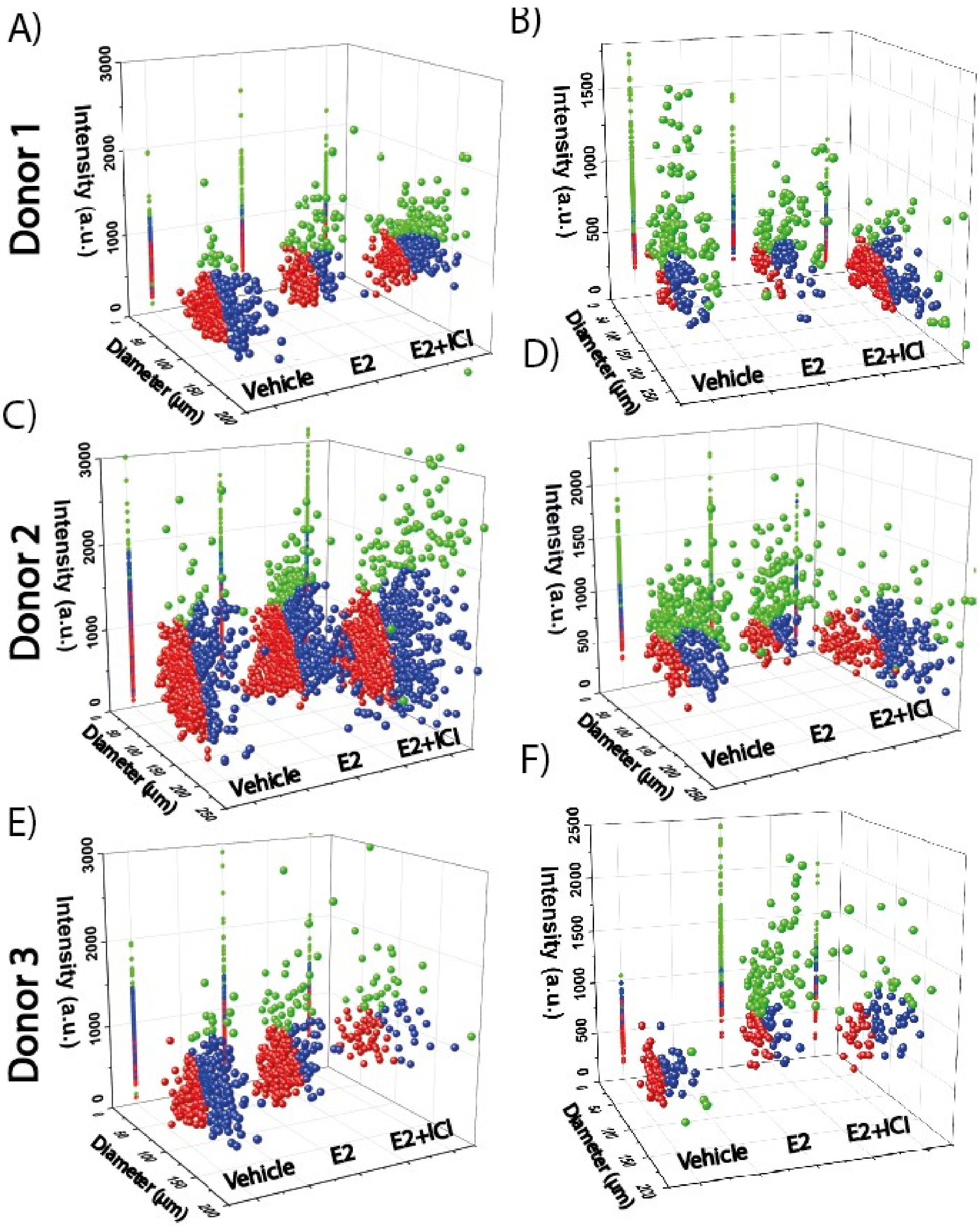

Figure S7. Organoids are labeled into three groups based on fluorescence intensity and area using supervised machine learning. *After the organoid labeling, each data point (organoid) will have assigned a label: L (red), I (blue), or H (green). The organoids are plotted as a function of Ki67 fluorescence intensity, diameter, and treatment condition. MCF7 labeled organoids with donor 1 (A), donor 2 (C), and donor 3 (E). ZR-75 labeled organoids with donor 1 (B), donor 2 (D), and donor 3 (G).*

Table S1. ASC donor characteristics

Donor	Age (years)	BMI	Derived
1	37	36.3	Abdomen
2	34	28.1	Abdomen
3	68	28.3	Abdomen

Table S2. Nomenclature system for each cluster. Each cluster was named as follows: Monoculture spheroids start with the name of the cell line, MCF7, ZR-75, or ASC followed by the cluster L (low), I (intermedia), or H (high). Organoids are referred to as M (MCF7) or Z (ZR-75) (depending on the BC cell line), followed by the donor number, and the cluster label.

Acronym	Cell line	Co-Culture with	Cluster
$MCF7^{L}$	MCF7	--	Low
$MCF7^{I}$	MCF7	--	Intermediate
$MCF7^{H}$	MCF7	--	High
$MDonor1^{L}$	MCF7	Donor 1	Low
$MDonor1^{I}$	MCF7	Donor 1	Intermediate
$MDonor1^{H}$	MCF7	Donor 1	High
$MDonor2^{L}$	MCF7	Donor 2	Low
$MDonor2^{I}$	MCF7	Donor 2	Intermediate
$MDonor2^{H}$	MCF7	Donor 2	High
$MDonor3^{L}$	MCF7	Donor 3	Low
$MDonor3^{I}$	MCF7	Donor 3	Intermediate
$MDonor3^{H}$	MCF7	Donor 3	High
$ZR\text{-}75^{L}$	ZR-75	--	Low
$ZR\text{-}75^{I}$	ZR-75	--	Intermediate
$ZR\text{-}75^{H}$	ZR-75	--	High
$ZDonor1^{L}$	ZR-75	Donor 1	Low
$ZDonor1^{I}$	ZR-75	Donor 1	Intermediate
$ZDonor1^{H}$	ZR-75	Donor 1	High
$ZDonor2^{L}$	ZR-75	Donor 2	Low
$ZDonor2^{I}$	ZR-75	Donor 2	Intermediate
$ZDonor2^{H}$	ZR-75	Donor 2	High
$ZDonor3^{L}$	ZR-75	Donor 3	Low
$ZDonor3^{I}$	ZR-75	Donor 3	Intermediate
$ZDonor3^{H}$	ZR-75	Donor 3	High
ASC^{L}	ASC	--	Low
ASC^{I}	ASC	--	Intermediate
ASC^{H}	ASC	--	High

Table S3. Average intensity and diameter per cluster in the organoids and spheroids. This table shows the average Ki67 fluorescence intensity and diameter in each of the clusters.

Cluster	Intensity (a.u)	Standard error	Diameter (µm)	Standard error
MCF7^{L}	488.0	10.5	89.0	1.1
MCF7^{I}	742.6	19.5	154.0	2.4
MCF7^{H}	1810.6	73.1	115.1	6.4
MDonor1^{L}	631.8	8.3	101.2	0.7
MDonor1^{I}	853.7	13.8	142.5	1.2
MDonor1^{H}	1308.1	24.8	127.5	2.2
MDonor2^{L}	776.8	8.9	115.5	0.7
MDonor2^{I}	1157.0	14.3	180.3	1.2
MDonor2^{H}	2219.9	127.5	161.5	3.2
MDonor3^{L}	668.3	12.1	93.6	1.0
MDonor3^{I}	927.6	16.2	141.0	1.2
MDonor3^{H}	1718.2	106.6	110.4	3.4
ZR-75^{L}	270.0	12.3	105.0	4.0
ZR-75^{I}	611.6	20.0	222.8	5.2
ZR-75^{H}	1143.5	54.3	195.1	10.8
ZDonor1^{L}	295.0	6.1	120.3	1.9
ZDonor1^{I}	464.2	9.4	187.6	2.6
ZDonor1^{H}	802.4	19.3	176.7	4.5
ZDonor2^{L}	536.3	9.4	109.5	1.8
ZDonor2^{I}	751.2	11.5	176.5	1.8
ZDonor2^{H}	1170.7	15.2	145.9	2.8
ZDonor3^{L}	565.6	16.4	87.2	1.4
ZDonor3^{I}	821.6	17.3	134.0	2.3
ZDonor3^{H}	1372.5	37.5	127.2	4.3
ASC^{L}	954.2	23.6	65.4	1.2
ASC^{I}	1811.2	100.1	114.5	2.0
ASC^{H}	1966.3	49.3	73.1	1.5

Table S4. Intensity and diameter per cluster separated by condition in the MCF7 organoids. This table shows the average Ki67 fluorescence intensity and diameter in each of the clusters separated by treatment condition.

Cluster	Condition	Intensity (a.u.)	Standard error	Diameter (μm)	Standard error
MCF^{L}	Vehicle	511.6	10.0	89.4	1.2
	E2	615.4	14.2	82.7	1.2
	E2+Fulv	438.7	9.0	90.0	1.1
MCF^{I}	Vehicle	712.8	16.6	159.9	2.4
	E2	873.0	24.7	157.6	2.3
	E2+Fulv	675.6	13.0	140.5	1.8
$MCF7^{H}$	Vehicle	1743.0	73.3	123.1	7.3
	E2	1813.1	73.1	114.8	6.3
	E2+Fulv	--	--	--	--
$MDonor1^{L}$	Vehicle	554.3	7.7	103.8	0.7
	E2	669.1	7.6	97.5	0.6
	E2+Fulv	717.4	7.4	100.2	0.6
$MDonor1^{I}$	Vehicle	743.9	15.3	150.5	1.4
	E2	900.8	9.4	133.4	0.9
	E2+Fulv	968.9	7.3	136.2	1.1
$MDonor1^{H}$	Vehicle	1102.7	32.4	118.8	3.0
	E2	1396.0	26.1	120.4	2.2
	E2+Fulv	1301.7	21.3	131.9	2.0
$MDonor2^{L}$	Vehicle	750.8	8.5	117.2	0.7
	E2	885.3	8.3	116.3	0.7
	E2+Fulv	681.6	8.6	113.4	0.8
$MDonor2^{I}$	Vehicle	1146.2	14.9	177.9	1.1
	E2	1240.8	11.0	175.8	1.4
	E2+Fulv	1087.6	15.8	185.7	1.1
$MDonor2^{H}$	Vehicle	2714.5	188.7	145.6	2.8
	E2	1817.7	20.9	143.9	2.4
	E2+Fulv	2475.1	157.5	182.3	3.4
$MDonor3^{L}$	Vehicle	594.4	10.9	103.4	0.9
	E2	670.3	12.3	90.1	0.9
	E2+Fulv	820.4	9.3	85.0	1.1
$MDonor3^{I}$	Vehicle	882.5	15.7	143.3	1.1
	E2	1005.8	15.0	132.8	0.9
	E2+Fulv	1144.0	12.4	138.7	1.4
$MDonor3^{H}$	Vehicle	1474.7	48.3	133.2	3.7
	E2	1773.7	129.6	103.6	2.7
	E2+Fulv	1768.8	93.5	107.8	3.5

Table S5. Intensity and diameter per cluster separated by condition in ZR-75 organoids. This table shows the average Ki67 fluorescence intensity and diameter in each of the clusters separated by treatment condition.

Cluster	Condition	Intensity (a.u.)	Standard error	Diameter (µm)	Standard error
$ZR\text{-}75^{L}$	Vehicle	274.0	11.3	93.8	3.7
	E2	397.7	15.2	105.5	3.7
	E2+Fulv	237.5	10.4	118.7	3.9
$ZR\text{-}75^{I}$	Vehicle	625.2	20.9	238.8	6.0
	E2	723.3	10.9	192.6	3.8
	E2+Fulv	566.4	18.9	216.7	3.9
$ZR\text{-}75^{H}$	Vehicle	917.9	36.8	167.6	9.0
	E2	1170.5	56.3	195.1	11.1
	E2+Fulv	1102.5	8.0	259.8	2.3
$ZDonor1^{L}$	Vehicle	349.9	5.0	121.8	1.7
	E2	379.1	4.6	108.9	1.7
	E2+Fulv	273.8	5.7	122.2	1.9
$ZDonor1^{I}$	Vehicle	511.7	7.1	187.6	2.6
	E2	537.2	7.0	176.4	2.6
	E2+Fulv	393.9	8.6	193.7	2.4
$ZDonor1^{H}$	Vehicle	865.2	20.7	173.8	4.5
	E2	790.9	15.0	172.5	4.0
	E2+Fulv	599.8	14.9	196.6	5.4
$ZDonor2^{L}$	Vehicle	597.3	7.6	109.0	1.5
	E2	637.0	6.3	107.0	1.2
	E2+Fulv	456.8	8.5	111.0	2.1
$ZDonor2^{I}$	Vehicle	820.1	8.9	175.1	1.5
	E2	823.6	5.2	146.1	0.8
	E2+Fulv	690.3	12.7	185.7	1.8
$ZDonor2^{H}$	Vehicle	1154.2	13.1	145.5	2.7
	E2	1209.9	17.1	133.4	1.9
	E2+Fulv	1116.4	17.2	192.4	3.9
$ZDonor3^{L}$	Vehicle	443.0	12.3	93.6	1.5
	E2	693.1	11.8	81.8	1.2
	E2+Fulv	582.7	15.4	84.6	1.3
$ZDonor3^{I}$	Vehicle	703.0	14.3	143.8	2.2
	E2	876.1	13.1	122.7	1.9
	E2+Fulv	875.8	16.6	134.1	2.4
$ZDonor3^{H}$	Vehicle	727.9	23.2	196.3	1.8
	E2	1403.7	37.4	120.7	4.3
	E2+Fulv	1366.3	26.1	143.9	2.7
